# Urban flooding as a biohazard risk: A call to address environmental and health inequities in tropical cities

**DOI:** 10.1371/journal.pntd.0013981

**Published:** 2026-02-17

**Authors:** Marco Coral-Almeida, Esteban Ortiz-Prado, Jorge Vasconez-Gonzalez

**Affiliations:** 1 Biochemoinformatics Group (GBQ), Universidad de las Américas, Quito, Ecuador; 2 One Health Research Group, Universidad de Las Américas, Quito, Ecuador; The University of Sydney School of Veterinary Science, AUSTRALIA

Urban flooding in tropical, low-income metropolitan areas such as Guayaquil (Ecuador), Salvador (Brazil), and Dar es Salaam (Tanzania) constitutes a chronic public health threat that has been largely underestimated in the neglected tropical diseases (NTDs) agenda. While the biological risks associated with flooding are well-documented in high-income settings, the compounding effects of poverty, inadequate sanitation, and informal urbanization in tropical regions render these events particularly hazardous.

Numerous factors have been described that contribute to the spread of diseases during floods, among which are:

- **Climatic factors:** Due to alterations in weather patterns, meteorological events such as floods are occurring with greater frequency and intensity. These events create breeding grounds for vector-borne diseases and contaminate water sources with pathogens [1].
- **Socioeconomic factors:** Communities living in poverty often face difficulties in accessing adequate housing, clean water, and healthcare services, increasing their vulnerability to infections. Furthermore, displacement caused by flooding exacerbates the spread of diseases [1].
- **Health system response:** The capacity, preparedness, and resilience of health systems, along with the effectiveness of flood preparedness and response plans, are crucial for improving disease management and reducing mortality rates [1].

Floodwaters in these contexts routinely carry a broad spectrum of pathogens including bacteria, viruses and parasites [2,3]. Common diseases associated with floods include waterborne diseases such as *Legionella*, *Cryptosporidium*, *Giardia*, *Escherichia coli*, *Salmonella*, and *Shigella*; soil-transmitted helminths such as *Ascaris lumbricoides*, *Trichuris trichiura*, *Ancylostoma duodenale*, and *Necator americanus*; and fungal infections such as chromoblastomycosis, blastomycosis, mucormycosis, and dermatophytosis [1]. Vector-borne diseases, especially mosquito-borne infections such as Dengue, Rift Valley fever, malaria, and West Nile fever, also tend to increase [4]. These microbes infiltrate communities through a complex network of compromised infrastructure open sewers, increase in vector breeding sites, unsealed drainage systems, and overflow from pit latrines frequently located in or near informal settlements [2,3].

Berendes and colleagues found that open drain flooding in urban neighborhoods of India increased the risk of pediatric enteric infections [5]. In Brazil, Reis and colleagues reported that residents living near open sewers were 42% more likely to contract leptospirosis during flood events, with each US$1 increase in daily income associated with an 11% reduction in risk [6]. These findings are mirrored in numerous cities across the global South, where rainfall turns streets into sewers and populations into passive recipients of pathogen exposure.

Despite this, flood-associated biohazard risks remain underrepresented in both policy discourse and intervention frameworks for NTDs. Recent systematic reviews and global climate–health analyses indicate that climate change is already reshaping the spatial and temporal patterns of flood-associated infectious diseases, including dengue, leptospirosis, and other NTDs. Projections linking rainfall intensity, flood duration, and altered urban hydrology with increased disease risk further underscore the urgency of integrating climate adaptation into NTD control strategies, particularly in rapidly urbanizing tropical regions [1,7–15].

While studies have consistently linked urban flooding to spikes in gastrointestinal illness, respiratory infections, and vector-borne diseases, health systems often fail to provide targeted surveillance or response mechanisms in informal settlements, particularly for waterborne diseases that thrive post-inundation [16]. Further research is needed to quantify and mitigate health impacts of floodings in tropical cities. This research could be done through epidemiological spatial-temporal studies linking both raining patterns and NTDs incidence, mathematical modeling coupling hydrological dynamics and disease transmission dynamics and intervention trials evaluating community-level mitigation strategies [17–19].

The intersectionality of environmental risk and socioeconomic vulnerability must be central to NTD policy and programming. Gender, age, and occupation influence not only who gets exposed, but also who can access timely care. As shown in the NTD gender equity literature, structural and behavioral constraints such as caregiving roles or social stigma disproportionately affect women’s and girls’ access to care during outbreaks of flood-associated diseases like leptospirosis or schistosomiasis [20].

To advance toward universal health coverage and the NTD 2030 goals, we must integrate flood preparedness and environmental justice into our approach to neglected tropical diseases. Public health strategies must include:

- Strengthening drainage and sanitation infrastructure in flood-prone urban areas: Strengthening drainage and sanitation infrastructure should include targeted upgrades of undersized or poorly connected stormwater networks, complemented by low-impact development and water-sensitive urban design solutions such as rain gardens, bioretention systems, and porous pavements. These approaches have been shown to reduce peak surface runoff and flood extent in tropical and monsoon-affected cities, including Cairns (Australia) and Gurugram (India) [21–24].
- Mapping biohazard hotspots for rapid NTD response: Mapping biohazard hotspots for rapid NTD response can be informed by spatio-environmental risk mapping approaches, such as the identification of leptospirosis risk around defective drainage systems, highly impermeable surfaces, and socially vulnerable neighborhoods, as demonstrated in Campinas (Brazil). In addition, emerging image-based and crowdsourced surveillance platforms piloted in Nigeria offer promising tools for the real-time detection of NTD hotspots in flood-prone urban settings [25–27].
- Deploying culturally competent health promotion targeting flood-exposed communities: Deploying culturally competent health promotion strategies can build on flood-preparedness education programs, such as the HEBI trial in Malaysia, which significantly improved community knowledge and response capacity. These initiatives should be complemented by best practices in climate-risk communication that tailor messages, messengers, and delivery channels to the needs of diverse urban populations [28–31].
- Integrating environmental health risk into surveillance systems and national NTD plans: Integrating environmental health risks into surveillance systems and national NTD plans can build on global analyses linking flood duration with increased incidence of dengue, leptospirosis, and other infections. These efforts can be further strengthened through geostatistical and machine-learning approaches that combine rainfall and environmental data with routine NTD surveillance to generate operational early-warning tools and dynamic risk maps [7,27,32,33].

Urban flooding in tropical cities is not merely an environmental nuisance; it is a recurring biological disaster. Recognizing and addressing this within the NTD community is not only a scientific imperative but a moral one.
